# The Diagnosis of Swellings in the Pelvis

**Published:** 1909-09-18

**Authors:** Arthur Giles

**Affiliations:** Surgeon to the Chelsea Hospital for Children, Gynæcologist to the Prince of Wales's Hospital, Tottenham


### Hospital Clinics.

### THE DIAGNOSIS OF SWELLINGS IN THE PELVIS.

By ARTHUR GILES, M.D., B.Sc., F.R.C.S.; Surgeon to the Chelsea Hospital for Children, Gynæcologist to the Prince of Wales's Hospital, Tottenham.

(A Clinical Lecture delivered at the Prince of Wales's Hospital, Tottenham.)

Gentlemen,—I have found it necessary to change the subject of my lecture. I had hoped to give you some of the results of removing the appendages, results collated from cases after operation, but the material was so extensive that I have not had time to work it up. So I propose to talk on a rather more practical subject, that of the diagnosis of swelling in the pelvis. One is apt to encounter such swellings at any time, often without warning, and I think we should run through the possibilities on some general scheme or plan; then the diagnosis will be a good deal easier. I shall go on the principle which I have already adopted in my little book on Gynæcological Diagnosis, namely, arguing from the known to the unknown. I shall not say, in talking of pelvic tumours, that the symptoms of fibroid, or ovarian tumour, are so and so. We are face to face with a tumour in the pelvis, and we want to know what it is.

Perhaps the most convenient way of beginning our classification will be to divide the cases into those that are definitely uterine, cases which are definitely not uterine, cases in which the condition is doubtful, and cases in which more than one tumour are present.

### Definitely Uterine Cases.

The first great group are the definitely uterine tumours. You may be able to discover this in various ways. You may be able to feel the appendages independently of the uterus and realise that they are healthy. Or you may make certain there is nothing in the pelvis corresponding to the uterus except the tumour, that is, that the whole thing moves *en bloc*.

The next thing to take as the clue is the condition of the patient's menstruation. If there is amenorrhœa this may be primary, that is to say, the patient has never menstruated at all, and yet there is this swelling in the pelvis. Of course, there are cases of atresia of the vagina, where you cannot get in, and it is only *per rectum* that you can find there is any swelling in the pelvis. If there is primary amenorrhœa and a swelling in the pelvis, with a patulous vagina, it is probable that the external os has no way through it; and the associated symptom which the patient will complain of will be, perhaps, some pain at more or less regularly recurring monthly intervals. So with these data before you, you can make a diagnosis of hæmatometra.

But the case may be one in which the patient has been menstruating regularly, but has ceased to do so for two or three months. When you get amenorrhœa following on normal menstruation, then in 99 per cent. of the cases you have to deal with pregnancy. When you investigate further you perhaps find the cervix soft and the vagina of a violet colour. To the feel, hæmatometra and pregnancy are very much alike, if the uterus is in the normal position. With the symptoms of pregnancy there may be associated symptoms of bladder trouble, first frequency of micturition, and then retention of urine. From these you can easily make your diagnosis of retroversion of the gravid uterus. The amenorrhœa, however, may not be absolute, but may be followed by irregular hæmorrhage or floodings. The uterus may not be as soft and elastic as in a normal pregnancy, but is rather harder and doughy. Then you will probably find that the cervical canal, instead of being normally closed, is partly patulous and dilated. And there are symptoms of pregnancy, in addition to which the patient probably has some pains in the abdomen of a bearing-down character, which she will tell you are like labour pains. You can then say it is pregnancy. But you can also say that abortion is either imminent or probable.

Then there is the second group, in which, instead of amenorrhœa, there is metrorrhagia, or menorrhagia, without any previous period of amenorrhœa. You are then practically certain to find the uterus large and hard; it may be smooth and uniformly enlarged, or it may be irregularly enlarged. There may be no symptoms except the fact of the hæmorrhage, although there may also be a history of pain, and perhaps of some bladder disturbance. On these data you are justified in diagnosing fibroid, including in that term pure fibroma, fibro-myoma, and fibro-adenoma. This applies equally to polypus inside the uterus. If it were a mucous polypus, no particular enlargement of the uterus would be noticeable.

### Pyometra with Cervical Cancer.

There is another form of enlargement of the uterus which is worth mentioning, where we should probably find neither amenorrhœa nor menorrhagia, but some irregular hæmorrhage, scanty in amount. An examination may show an enlargement of the uterus, associated with carcinoma of the cervix, with sometimes a rise of temperature and more pain than is usual with carcinoma of the cervix. When in such a case you have said carcinoma of the cervix, your diagnosis is not complete; it is almost certainly something more, namely, pyometra. That is comparatively rare, except in association with carcinoma of the cervix. The reason is that the growth in the cervix obliterates the cervical canal, and being more or less a septic growth it is liable to lead to an accumulation of pus inside the uterus.

Carcinoma of the cervix by itself never causes marked enlargement of the uterus such as would justify its inclusion under the heading of pelvic tumours. Of course, it may also have been pregnancy, but we are not considering that just now. A point of some interest is, that you will hardly ever find carcinoma of the uterus associated with early pregnancy, the reason being that conception is almost impossible when there is carcinoma of the cervix present. When you find the two conditions present, nearly always the pregnancy has taken place before the carcinoma has made much advance.

### The Group of Non-uterine Tumours.

The next group is where we are certain that the tumour is not uterine. Here again it is useful to begin by inquiring into menstruation. If there is amenorrhœa associated with a tumour which is distinct from the uterus—unless at the menopause—it is practically certain to be extra-uterine pregnancy. The tumour may be mobile, or it may be fixed. Suppose it is mobile. With these facts you will almost certainly find the amenorrhœa is of short duration, not more than two or three months, and the tumour which is present will be an unruptured tubal pregnancy. One might include ovarian pregnancy, but that is so rare that we need not mention it specifically.

Of course, these cases are not often diagnosed. I have not seen more than two or three cases that I have diagnosed as unruptured extra-uterine pregnancy, and probably it has not fallen to your lot to do it very often either. The reason is that when extra-uterine pregnancy is unruptured, as a rule there are no symptoms at all. The patient thinks it is an ordinary pregnancy, and therefore there is nothing to call attention to the pelvis. And if a woman comes to you and asks you to attend her in seven months' time, you do not think it necessary then to examine her. Of course, I do not say it would be a bad thing if examination were made at that early period; no doubt it would be sometimes inconvenient, but it would save many difficulties afterwards.

### Fixation and Mobility.

But suppose the tumour is fixed and there is amenorrhœa, you will almost always find that there has been a little trickling hæmorrhage, an irregular, scanty, blood-stained discharge. And the diagnosis you must make then is ruptured tubal pregnancy. A mobile tumour under these conditions is generally on one side of the uterus; occasionally it may be in front, or behind. A fixed tumour is most often on one side, but it may be behind. If on one side, it is almost certainly in the broad ligament itself. If it is behind, it generally means that there has been a rupture into the general peritoneal cavity, that blood has accumulated in the pouch of Douglas, and has formed a pelvic hæmatocele.

The fact that a tumour of this sort is definitely fixed, is rather useful from the point of view of diagnosis. I may tell you of a case which happened to me ten or twelve years ago, when, of course, I had had less experience. I saw a patient who was complaining of pain on one side, and of a little irregular hæmorrhage. On examining, I thought I found a tumour to the left of the uterus, and on those data I said I thought it was extra-uterine pregnancy. I noticed I could push up this tumour, but in spite of that I still thought it was extra-uterine pregnancy, but I did not feel quite sure enough to advise operation, so I said we would wait. I saw her a fortnight later, and by this time the tumour at the side had shifted, and was definitely the fundus of the uterus. She was still having a little hæmorrhage, and we concluded she was in for a miscarriage, and she verified the diagnosis a few weeks later. My error arose from not taking sufficient notice of the fact that if it was ruptured it must be fixed. If it is a mobile extra-uterine pregnancy which is unruptured, there will be no hæmorrhage, and if it is ruptured with hæmorrhage, it will be fixed.

### Lateral Tumours with Unaltered Menstruation.

We come now to a larger group, in which the menstruation is unaltered, and there is a tumour at the side of the uterus. We can determine at once whether it is mobile or fixed. With a mobile tumour by the side of the uterus with menstruation unaltered you can at once exclude pregnancy, and therefore it must be some form of new growth. It could not be inflammatory, or it would not be mobile. So you can at once say it is new growth of some sort. According to its position you will be able to get a little further.

With a mobile tumour by the side of the uterus, rather cystic and rounded, you will be pretty safe in diagnosing a small ovarian. If in a similar position the tumour be very hard, and closely connected with the uterus, you can diagnose pedunculated fibroid. I say small ovarian, because a large ovarian would never be by the side of the uterus. When it is bigger, it either gets jammed behind the uterus and causes symptoms of impaction, or, what is more usual, it gets in front of the uterus, and presses the uterus back into the hollow of the sacrum, and as the ovarian gets larger still, it rises up in the abdomen, releases the uterus, and lies above it. Later, when the tumour is too large to sink into the pelvis and is abdominal, the position of the uterus may be normal.

But there are a few other things which are worth bearing in mind. There might, for instance, be a mobile swelling to the left of the uterus, not rounded and cystic, nor even hard, but rather irregular, and of medium consistency, perhaps doughy. Such a tumour may be a growth of the sigmoid. It is too high up to be reached by the rectum. And besides, if it were of the rectum it would be fixed; but where the meso-sigmoid allows of movement you may get a growth there which rather closely resembles some of these things. That growth is practically certainly carcinoma. It would have to be on the left side. There might be on the right side a growth of the cæcum, but that is much less likely, for the reason that it is very rare for the cæcum to occupy a position in the pelvis. The sigmoid comes into the pelvis below the brim, but the cæcum hardly ever does.

One should mention the fact that sometimes one can feel a mobile swelling by the side of or behind the uterus, or in front, softer than any we have mentioned so far, and definitely elongated. With such a tumour I recommend you to be guarded in your diagnosis, and say you would like to see the patient in a week's time, and give her purgatives, because in thin people it is possible to feel distended coils of intestine. There may be a swelling in front of the uterus, which moves easily, as if it were in fluid; that is certain to be a foreign body in the bladder.

### Fixed Separate Tumours.

Now let us take the second division, in which the tumour is fixed, still with menstruation unaltered, and a separate tumour. These fixed tumours I think we should take according to their position. Take first the lateral ones. What are the swellings which we may find fixed by the side of the uterus with unaltered menstruation? The tumour may be globular and cystic, and give all the feeling of an ovarian cyst. If a cyst be fixed it must be for some reason, either because inflammation around it has caused it to adhere, or because it is impacted, or because of its developing in the broad ligament, which also prevents it rising. The cyst in the broad ligament would give the fewest symptoms; indeed, it might develop without causing any symptoms beyond a little fulness or a slight aching in the pelvis. But if there is inflamed ovarian, there will be definite pain and tenderness, and with impacted ovarian cyst there would be symptoms of pressure.

Instead of being spherical or globular, this lateral cystic swelling may be ovoid or irregular. Probably it is not a true cyst, for a true cyst always tends to be spherical: it is fluid in the tubes, and so you have to do with hydrosalpinx. That may develop without any special pain, and without any particular symptoms. But there may be this same sort of feeling and swelling in a rather irregular body, associated with very marked pain, and perhaps with rise of temperature. Then you would be justified in supposing that the contents of that tube were not clear fluid, but pus, and so you would make the diagnosis of pyosalpinx.

The tumour may not be cystic at all, but may be hard. If it is a hard rounded tumour, what is most likely is that it is a fibroid of the uterus developing in the broad ligament, or fibroid of the broad ligament itself. But most fibroids originate in the uterus. Such a tumour which is hard may not be rounded and may not suggest fibroid. It may be irregular and give the well-known board-like hardness which you sometimes find in connection with pelvic inflammation, and then there will also be rise of temperature, and perhaps even rigors, and so you can say it is pelvic cellulitis. You know the feel; everything about the uterus seems involved in one hard mass. And you may get a pelvic abscess with the formation of pus developing in the broad ligament, where you cannot elicit fluctuation, because there is too much thickening at the base of the broad ligament between you and the pus. But it is a further possibility. You cannot, as a rule, tell which it is until you open up the region.

If there were marked rigors and a very up-and-down temperature suggestive of pus, you would very likely diagnose abscess; and if there were a temperature, not so high, but more sustained, with the patient not looking so ill, you would be more likely to diagnose pelvic cellulitis.

There may be a mass on the left side which is neither cystic nor very hard, but may be doughy where the tumour is carcinoma of the rectum. You may be able to feel the appendages separately. The broad ligament feels soft, and then, of course, you put your finger into the rectum and ascertain if there is a growth there. If there is any doubt, that is a thing which is worth doing; it may save you retracting your opinion afterwards, which may be an awkward matter, if you say it is probably ovarian and can be removed. "When in doubt say nothing"; and that may be interpreted by the patient as wisdom.

You may find a swelling behind the uterus, and those which occur there are virtually the same as those which you find at the side of it. There may be impacted ovarian, inflamed ovarian, impacted fibroid, or a pelvic inflammation, with corresponding symptoms and signs to those we have considered, so we need not go over them again.

### Fixed Anterior Swellings.

Then there are fixed swellings in front of the uterus. The most frequent is a hard rounded swelling, which is a fibroid developing from the anterior wall of the cervix, and pushing up the bladder. Its position causes it to be fixed. In front of the uterus there may also be a fixed cystic swelling. That practically cannot be ovarian, because an ovarian in front would have plenty of room in which to move and would not be fixed. And that reduces you to only one other thing, and that is a distended bladder. Some time ago a friend of mine wrote asking me to hold myself in readiness to go down and operate in a case of supposed rather large ovarian cyst. Two days later, however, he wrote and said that the passing of a catheter had resulted in 80 ozs. of urine coming away and the cyst had disappeared, and all was peace. He did not say what he told the patient after making the first diagnosis.

Thirdly, the swelling in front of the uterus may be a firm sort of swelling, almost hard, and rather irregular. In such a case there may be no symptoms except pain, or there may be some hæmorrhage on micturition. And if you pass a cystoscope you will almost certainly see there is carcinoma of the bladder. Without the cystoscope you may not be able to do more than infer that it is carcinoma of the bladder.

### The History of Three Unusual Cases.

Occasionally you meet with things which are absolutely out of the ordinary course of experience, and I will tell you the history of three cases somewhat on these lines. The first was a patient who was brought in here about sixteen months ago, with a history of having passed fæcal matter from the bladder. We cystoscoped her, and found merely a red, ulcerated-looking patch on the posterior wall of the bladder. We could feel there was a swelling to the left of the uterus, and I diagnosed an abscess in the pelvis communicating on the one hand with the bladder, and on the other hand with the bowel. We opened the abdomen, and found this swelling on the left of the uterus, which turned out to be a small suppurating ovarian cyst, full of pus, but independent of the other conditions. The sigmoid was adherent to the bladder direct, the uterus being turned down and pushed out of the way. On separating the sigmoid from the bladder there was a hole in each. It was in a woman æt. 28, and as I thought it was tuberculous I resected 4 in. of the bowel. Subsequent report showed it to be carcinoma of the sigmoid, adherent to and ulcerating into the bladder. She is all right, although it is sixteen months since the operation.

The second case was that of a patient who had been passing fæcal matter in considerable quantities by the bladder. Again it was carcinoma of the sigmoid, but associated with extensive carcinoma of the bladder, so that any question of removal had to be put on one side. I should think such a combination is very rare; yet three weeks after seeing that case I saw another almost precisely similar in regard to the history, and on opening up I found carcinoma of the bowel and bladder, and in that case it had affected the bladder in a curious way. The growth had spread round the base and lower part of the sides of the bladder, so that it made a complete hard cup, and as the bladder was empty at the time of the operation the fundus of the bladder dropped into this hard cup, and produced that curious appearance.

For the sake of symmetry we may put a third group, that with menstruation increased, say menorrhagia; but that would have comparatively little diagnostic value.

There are three conditions of tumour in this region apart from the uterus that may cause menorrhagia. The first is fibroids, and that presupposes that in addition to the tumour which you feel separate from the uterus, there must be a small one in the uterus causing menorrhagia. The second is carcinoma of the ovary. Ordinary ovarian tumour does not cause menorrhagia, but a carcinoma of the ovary may. Thirdly, there may be pus tubes, and without any alteration of menstruation, but it is not infrequent with pus tubes to find menstruation is increased.

### Doubtful Uterine Cases.

I said there was a third group in which one could not say for certain whether the tumour was uterine or not. There we have a comparatively easy way of settling the matter. If we find that menstruation has gone on unaltered we can at once exclude pregnancy, consequently we can pass a sound and see whether it goes into the tumour or not, and settle whether it is a uterine or a non-uterine tumour. If we find there is amenorrhœa we have only to decide whether it is a case of extra-uterine pregnancy or intra-uterine.

If the uterus and tumour are in a normal position we may at once feel certain it is not extra-uterine pregnancy. If the tumour is by the side of or behind the uterus we shall soon conclude whether it is uterine or not; because we should only have to decide whether we had to do with retroversion of the gravid uterus, or an extra-uterine pregnancy. Time will decide that.

Retroversion of the gravid uterus will probably not be associated with hæmorrhage at all, unless the case is one of threatened abortion, whereas a tumour situated behind the uterus simulating retroversion of the gravid uterus would be a ruptured uterine, and there would almost certainly be hæmorrhage.

### Multiple Tumours.

And we have left the cases in which there is more than one tumour. In those it is not a question of diagnosis but of guessing. I shall not detain you with details now. You can practically settle for yourselves the permutations and combinations: there may be pregnancy with fibroid, with ovarian, with pus tubes, with extra-uterine pregnancy; there may be fibroids with the same combinations; there may be multiple fibroids and multiple ovarians, or there may be a double uterus.

And in addition to all these possible conditions you may have a growth in connection with the sigmoid or rectum as well, and those are things which no man can diagnose with any certainty. If you do make a correct diagnosis, you may consider you are at least as lucky as you are clever. I operated in one case where I thought I should find multiple fibroids. There were fibroids in the uterus, but the main mass was a large suppurating ovarian cyst on the right side, in the walls of which there were carcinomatous deposits with other deposits on the back of the uterus. Nothing short of inspiration would enable one to diagnose a condition of that kind. Such a case has always to be investigated by opening the abdomen.

